# Protective Effect of Vaginal *Lactobacillus paracasei* CRL 1289 against Urogenital Infection Produced by *Staphylococcus aureus* in a Mouse Animal Model

**DOI:** 10.1155/2007/48358

**Published:** 2007-03-29

**Authors:** Gabriela Zárate, Viviana Santos, María Elena Nader-Macias

**Affiliations:** ^1^Departamento de Microbiologia Preventiva, Centro de Referencia para Lactobacilos (CERELA)-CONICET, Chacabuco 145, 4000 San Miguel de Tucumán, Tucumán, Argentina; ^2^Facultad de Bioquímica, Química y Farmacia, Universidad Nacional de Tucumán, Tucumán, Argentina

## Abstract

Urogenital infections of bacterial origin have a high incidence among the world female population at reproductive age. Lactobacilli, the predominant microorganisms of the healthy vaginal microbiota, have shown a protective effect against the colonization and overgrowth of urogenital pathogens that increased the interest for including them into probiotics products assigned to restore the urogenital balance. In the present work, we determined in a mouse animal model the capability of *Lactobacillus paracasei * CRL 1289, a human vaginal strain with probiotic properties, to prevent the vaginal colonization of a uropathogenic strain of *Staphylococcus aureus*. Six-week-old female BALB/c mice, synchronized in their estral cycle, were intravaginally inoculated with two doses of 10^9^ lactobacilli before challenging them with a single dose of 10^5^ or 10^7^ CFU of *S. aureus*. The vaginal colonization of both microorganisms and the effect on the vaginal structure were determined at 2, 5, and 7 days after pathogen inoculation. Control mice and those challenged only with the pathogen showed an insignificant lactobacilli population, whereas 10^5^ lactobacilli/mL of vaginal homogenate were recovered at 2 days after challenge from the *L. paracasei* CRL 1289 and the probiotic + pathogen groups, decreasing this number on the following days. The treatment with *L. paracasei* CRL 1289 decreased significantly the number of staphylococci recovered at 2 and 5 days when mice were challenged only with 10^5^ CFU of pathogen. The inoculation of *S. aureus* produced a remarkable inflammatory response and structural alterations in the vaginal mucosa that decreases in a significant manner when the mice were protected with *L. paracasei* CRL 1289. The results obtained suggest that this particular *Lactobacillus* strain could prevent the onset of urogenital infections by interfering with the epithelial colonization by uropathogenic *S. aureus*.

## 1. INTRODUCTION

Urogenital tract infections of bacterial origin have a high incidence among the world female population at reproductive age. A great proportion of these diseases, such as vaginosis and urinary tract infections are often caused by pathogens that emerge from the intestinal microbiota and ascend along perineum to the vagina and then to the urethra and bladder [1]. While antibiotics have been extensively used as a quite effective therapy for the treatment of these bacterial infections, the increasing drug resistance of urogenital pathogens makes imperative the development of alternative therapeutics.

In healthy women, the vaginal microflora is dominated by *Lactobacillus* species, at a level of 10^7^-10^8^ CFU g^−1^ of fluid, which exert a significant influence on the microbiology of the ecosystem [2]. It has been observed that indigenous lactobacilli prevent the overgrowth and invasion of pathogenic bacteria [3] by a combination of competitive exclusion, competition for nutrients, and release of antimicrobial substances such as hydrogen peroxide, organic acids, bacteriocins, and biosurfactants [3–6]. In consequence, a depletion of vaginal lactobacilli has been directly associated with an increase in the incidence of genital and urinary infections [7–9]. For this reason, there is a growing interest in the use of human lactobacilli as probiotics that restore and maintain a normal vaginal flora and prevent disease recurrence by forming a pellicle on the vaginal epithelium as a biological barrier against colonization of pathogenic bacteria. In this sense, previous studies have reported that adhesive lactobacilli can inhibit in vitro the attachment of pathogens such as *Escherichia coli, Gardnerella vaginalis, Candida albicans, Pseudomonas aeruginosa, Klebsiella pneumoniae, Staphylococcus aureus*, and *Streptococcus agalactiae* to urogenital epithelial cells [5, 10–13].

Having in mind the objective of developing a probiotic formulation for the prevention and therapy of urogenital tract infections, our research group has previously isolated and identified vaginal lactobacilli from healthy women of Tucumán city in Argentina [14]. The strains were extensively characterized for their probiotic and technological features and some promising properties such as adhesion, auto and coaggregation abilities, hydrogen peroxide, bacteriocin-like substances, and organic acids production were reported [15–18]. Relevant technological properties, for instance, the optimal conditions for the production of antimicrobial substances and the viability and biological properties after processing, were also determined for selected strains [19–24].

*Lactobacillus paracasei* CRL 1289 is a selected human vaginal strain selected by its probiotic potential, since it is able to inhibit the growth of uropathogenic *Staphylococcus aureus* in vitro by release of H_2_O_2_ [20], and its adhesion to vaginal epithelial cells by exclusion and competence for specific receptors [24]. *Staphylococcus aureus* is a major opportunistic pathogen that can cause a variety of local and systemic infections ranging from skin abscesses, bone and soft tissue surgical infections, sepsis, invasive endocarditis, and toxic shock syndrome (TSS) [25]. TSS is a geographically widespread disease affecting mainly young healthy menstruating women, especially those using tampons [26].

The aim of the present work was to determine, in a mouse animal model, the capability of *Lactobacillus paracasei* CRL 1289 to prevent the vaginal colonization of uropathogenic *Staphylococcus aureus*.

## 2. MATERIALS AND METHODS

### 2.1. Microorganisms and growth conditions

*Lactobacillus paracasei* CRL 1289 was originally isolated from vaginal smears of healthy women [14] and was previously characterized by their probiotic and technological properties [15, 20, 22–24]. The human uropathogenic strain of *Staphylococcus aureus* used in this study was kindly provided by the Institute of Microbiology “LuisVerna” of the University of Tucumán, Argentina, and was isolated from pathological urine. Before experimental use, each strain stored in milk-yeast extract at −20°C was propagated in LAPTg broth (1.5% peptone, 1% tryptone, 1% glucose, 1% yeast extract, and 0.1% Tween 80, pH 6.8) [27] at 37°C and subcultured at least twice in this media every 12 hours. Lactobacilli were cultivated under static conditions in order to avoid the detrimental effects of oxygen whereas staphylococci were incubated with shaking at 100 rpm.

### 2.2. Animals

Six-week-old female BALB/c mice from the inbreed colony of CERELA (Centro de Referencia para Lacobacilos), each weighing from 25 to 30 g, were used throughout the investigation. Animals were housed in plastic cages and fed ad libitum with a conventional balanced diet, keeping their environmental conditions constant. All the animals were synchronized in their estrous cycle with an intramuscular single dose of 0.5 mg of estradiol valerate (Progynon Depot. Schering Laboratories) and randomly assigned to the following experimental groups: (1) lactobacilli treated group, (2) lactobacilli + pathogen treated group, and (3) pathogen treated group. Five animals were used as control synchronized nontreated group. The CERELA Committee of Ethics approved the protocol used for animal studies.

### 2.3. Microorganisms inoculation procedure

**Figure 1:**
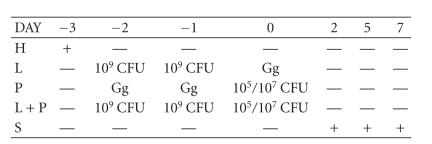
Scheme of inoculation of *L. paracasei* CRL 1289 and *Staphylococcus aureus* on Balb/c mice. H: hormone (estradiol valerate), L: lactobacilli inoculation, P: pathogen inoculation, L + P: lactobacilli plus pathogen inoculation, S: sacrifice of animals, and Gg: glycogelatin ovules without microorganisms.

A spontaneous rifampicin resistant strain obtained by plating *L. paracasei* CRL 1289 on Rogosa agar (Merck) supplemented with 150 *μ*g/mL of rifampicin was used to inoculate mice. The resistant strain showed exactly the same properties of the original strain. Overnight cultures of lactobacilli and staphylococci grown on Laptg broth (12 hours, 37°C) were centrifuged (10 000 g, 10 minutes, 4°C), washed twice with sterile saline solution, and incorporated into glycogelatin ovules at a concentration of 10^9^ CFU of lactobacilli and 10^5^ and 10^7^ CFU of *S. aureus* per each ovule. The base preparation of the ovules was obtained by mixing 21% gelatin and 58% glycerol in distilled water. This matrix was sterilized at 121°C for 15 minutes, and supplemented with 0.5% ascorbic acid and the suspensions of microorganisms in a proportion 1 : 5. Forty-eight hours after estradiol synchronization, animals of groups 1 and 2 were intravaginally inoculated with two doses of 10^9^ lactobacilli (with a 24-hour interval between each other). On the third day, the animals of group 2 and those belonging to group 3 were challenged with a single dose of 10^5^ or 10^7^ CFU of uropathogenic *S. aureus*. Figure 1 shows the inoculation protocol used.

### 2.4. Bacterial counts in vaginal homogenates

At 2, 5, and 7 days after pathogen inoculation, the animals were sacrificed by cervical dislocation. The vagina of each animal was removed aseptically, placed in 0.5% peptone-water, and homogenized with a Teflon pestle. Serial ten-fold dilutions from this homogenate were plated on Rogosa agar (LBS, Merck); Rogosa agar supplemented with 150 *μ*g/mL of rifampicin and Manitol Salt Agar (MSA, Britania) for counts of lactic acid bacteria, *L. paracasei* CRL 1289, and *S. aureus*, respectively. The LBS plates were incubated 72 hours under microaerophilic conditions whereas MSA plates were aerobically incubated.

### 2.5. Cytological and histological studies

The cytological and histological evaluations were carried out by light microscopy. For cytological studies, 50 *μ*L of vaginal exudates were collected with a micropipette tip, spread onto glass slides, and stained with Giemsa. For the study of histological structures, the vaginas were aseptically removed, fixed with 10% paraformaldehyde for 24 hours at room temperature, and then embedded in paraffin according to standard histological methods [28]. Serial paraffin sections of 4 *μ*m were stained with hematoxylin-eosin and observed at 40X.

### 2.6. Statistical analysis

The results are expressed as the mean value ± standard deviation of the data obtained from three animals at each sample time of two independent experiments. Significant differences between means were determined by Tukey's test after analysis of variance (ANOVA) with Minitab Statistic Program, release 12 for Windows. A *P* value of <.05 was considered statistically significant.

## 3. RESULTS

**Figure 2:**
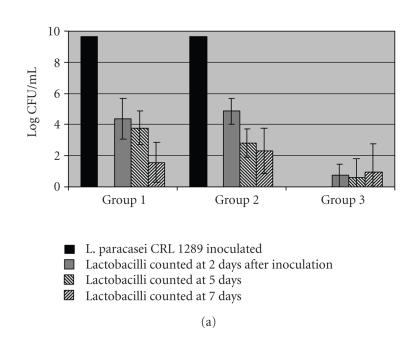

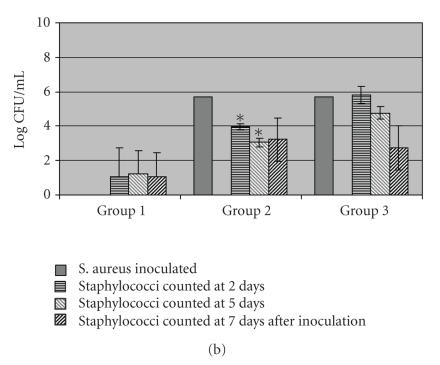
Viable counts of lactobacilli (panel A) and staphylococci (panel B) on the vagina of mice inoculated only with these microorganisms or pretreated with *L. paracasei* CRL 1289 and challenged with uropathogenic *S. aureus*. Panel A: (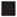) *L. paracasei* CRL 1289 inoculated, (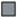) lactobacilli counted at 2 days after inoculation; (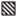) lactobacilli counted at 5 days; (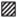) lactobacilli counted at 7 days. Panel B: (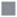) *S. aureus* inoculated; (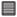) staphylococci counted at 2 days; (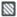) staphylococci counted at 5 days; (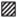) staphylococci counted at 7 days after inoculation.

Figure 2(a) shows the viable counts of lactobacilli and Figure 2(b) those of staphylococci on the vaginal homogenates of Balb/c mice at different sampling times after inoculation of probiotic *L. paracasei* CRL 1289 and uropathogenic *S. aureus*. Synchronized nontreated mice and those challenged only with the pathogen (Group 3) showed an insignificant lactobacilli population. On the other side, 10^5^ lactobacilli/mL of vaginal homogenate were recovered from the group inoculated only with probiotic *L. paracasei* CRL 1289 (Group 1) and the probiotic + pathogen group (Group 2) at 2 days after challenge. However, this number decreased progressively on the following days (see Figure 2(a)).

Referred to the number of pathogens recovered from mice, control group and the lactobacilli treated group (Group 1) were almost depleted of staphylococci population (Figure 2(b)). The inoculation of 10^7^ CFU of *S. aureus* produced a high and constant colonization of the pathogen that was not prevented by the pretreatment with lactobacilli (results not shown). However, the treatment with *L. paracasei* CRL 1289 previous to the pathogen infection decreased significantly the number of staphylococci recovered at 2 and 5 days when mice were challenged with 10^5^ CFU of the pathogen (Figure 2(b)).

**Figure 3:**
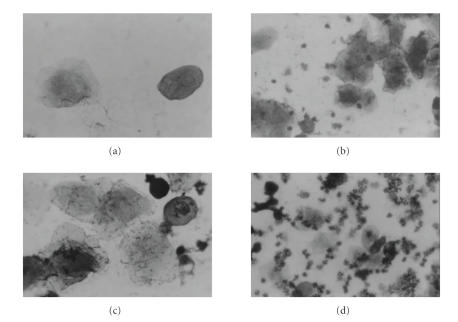
Vaginal smears stained with Giemsa of mice that were inoculated with lactobacilli and/or staphylococci. (a) Lactobacilli treated group; (b), (c) lactobacilli + pathogen treated group; (d) pathogen treated group. Magnification is 40X.

Figure 3 shows the vaginal smears stained with Giemsa of mice inoculated with lactobacilli and staphylococci. No cytological modifications were observed after the administration of *L. paracasei* CRL 1289. By the contrary, the inoculation of *S. aureus* produced a remarkable inflammatory response (Group 3) with a high infiltration of polymorphonuclear cells in the vaginal secretions. This effect decreased in a significant manner when mice were previously protected with *L. paracasei* CRL 1289.

**Figure 4:**
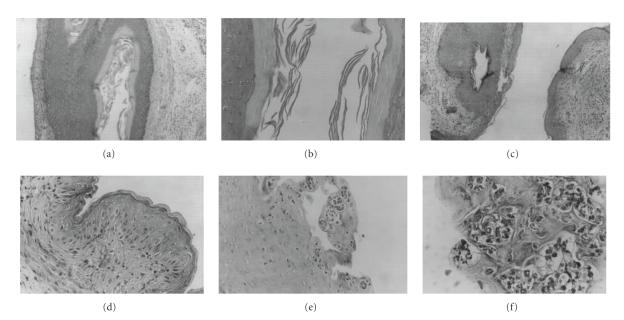
Light microscopy photographs of histological slices stained with hematoxylin-eosin showing the mucosa structure of the vaginas of mice that were inoculated with lactobacilli and/or staphylococci. (a), (b) Lactobacilli treated groups at 10X and 100X, respectively. (c), (d) Lactobacilli + pathogen treated groups at 40X and 100X. (e), (f) Pathogen treated groups at 40X and 100X magnifications, respectively. For details see Materials and Methods.

No histological alterations were produced by the lactobacilli inoculation (Figure 4), whereas significant structural modifications of the vaginal mucosa, with disappearance of the keratin layer and neutrophiles infiltration in the epithelium, were observed in the group inoculated solely with *S. aureus* (Figure 4). An intermediate effect was observed in mice protected with *L. paracasei* CRL 1289.

## 4. DISCUSSION

The potential use of human lactobacilli as probiotics assigned to restore and maintain a healthy urogenital tract represents a promising alternative to conventional chemotherapy [6, 29]. At present, a lot of scientific evidence supports, by in vitro and in vivo studies, the effectiveness of probiotics to prevent the attachment or stimulate the removal of enteropathogens from intestinal cells [30, 31]. Probiotics have been successfully used to prevent and treat gastrointestinal diseases caused by antibiotics treatments, rotavirus, enterobacteria and clostridia infections [32]. However, there are much lesser antecedents on the preventive and therapeutic effects of probiotics against diseases of the urogenital tract. Some in vitro studies have reported the inhibition of pathogens growth and adherence to uroepithelial cells by lactobacilli [10, 12, 13, 21, 24, 33]. This “anti-infective” mechanism involves the release of antimicrobials and the blockage of uropathogens adherence by both steric hindrance and competition for receptors [10, 34]. With respect to in vivo studies, it has been reported that vaginal lactobacilli prevented urinary tract colonization of mice by *E. coli* but were not able to exert significant therapeutic effects [35]. In humans, clinical efficacy for urogenital health maintenance and disease prevention has been demonstrated only for *Lactobacillus rhamnosus* GR-1 and *Lactobacillus reuteri* RC-14 [1]. Both probiotic lactobacilli strains colonized the urogenital tract by vaginal instillation and oral consumption, and were able to reduce the risk of UTI and cure bacterial vaginosis [36, 37].

In the healthy urogenital tract of adult females, it is supposed that the indigenous lactobacilli exclude the colonization of pathogenic bacteria by antagonistic compounds and/or by occupying/masking their potential binding sites in the mucosa [10, 38]. However, in a depleted lactobacilli environment such as an infected urogenital tract, it should be expected that exogenous probiotic lactobacilli have the capacity to compete for the same receptors and displace previously attached pathogens [11]. In a previous study, we observed that selected vaginal lactobacilli interfered to different extents with the growth and adherence to vaginal epithelial cells of some genitourinary pathogens [21, 24]. Among these strains, *Lactobacillus paracasei* CRL 1289 was able to decrease, in a significant level, the adhesion of *S. aureus* by exclusion and competition mechanisms [24], as well as to inhibit its growth by H_2_O_2_ production [15, 20]. Based on these findings, we evaluated in the present work the ability of these lactobacilli to prevent the vaginal colonization of *S. aureus* in an animal model, and the protection achieved.

The results obtained showed that lactobacilli were not a dominant population of the vaginal microbiota of the Balb/c mice used in this study and that human *Lactobacillus paracasei* CRL 1289 was able to colonize transiently the murine vaginal tract, since 10^5^ lactobacilli/mL of vaginal homogenate were recovered after 2 days of inoculation but decreased progressively on the following days. However, the human *S. aureus* uropathogenic strain was able to produce a very strong infection when inoculated at 10^5^ or 10^7^ CFU levels, producing significant morphological alterations of the mucosal structure, mainly the infiltration of polymorphonuclear cells that appeared as capsulated groups of cells in the vaginal epithelium and lamina propia; and the complete disappearance of the keratin layer. *Lactobacillus paracasei* CRL 1289 was not able to protect mice challenged with 10^7^ CFU of *S. aureus* but effectively decreased the number of staphylococci in the vagina and the damage caused, when the infecting dose of the pathogen was 10^5^ CFU.

In conclusion, the preliminary results obtained in this work suggest that *L. par*acasei CRL 1289 could prevent the onset of urogenital infections caused by uropathogenic *S. aureus* interfering with the epithelial colonization (possibly through barrier/interference mechanisms) and encourage further in vivo studies, such as clinical trials designed to test their capacity to prevent and manage urogenital tract infections in females.
